# Chronic Intestinal Stasis

**Published:** 1920-03-27

**Authors:** 


### CHRONIC INTESTINAL STASIS.

### New Discoveries in this Disease.

Chronic intestinal stasis, a condition productive of alimentary toxæmia, was first described in detail by Sir Arbuthnot Lane, and was a subject discussed at Societies, and written about very freely in the years 1913-1915. At that time great controversy existed around this subject; the disciples of Lane were quite sure of their ground, and the more conservative elements of the profession were equally sure that chronic intestinal stasis was a myth, and that Sir Arbuthnot Lane, the pioneer of so many surgical triumphs, had discovered a "mare's nest." The attitude of the great mass of the profession was a somewhat bewildered one, but on the whole comparable to that of the Scottish judicial verdict of "Non Proven," inasmuch as most medical men saw a germ of truth in the subject, even though not prepared to accept the full teaching of Lane and his followers. Like many other matters, it was more or less shelved during the Great War, but has now been revived by a most interesting paper read before the Manchester Medical Society by Mr. J. W. Smith, M.Ch., F.R.C.S., and entitled "Atony and Prolapse of the Large Intestine."

### Prolapse of the Large Intestine.

In cases of definite delay of the ileal effluent into the cæcum, there is nearly always a varying amount of actual prolapse of the large intestine; in some cases this is extreme, the whole colon lying almost below the level of the pelvic brim, with the exception of the splenic flexure. The colon seems to have its maximum fixity in the region of the spleen, and this fastening prevents prolapse of this particular portion of the intestine; so much so, that the transverse colon may ascend almost vertically from the pelvis and form a very acute angle with the descending colon.

Various "bands" and "membranes" are found in relation to the colon and lower ileum even in otherwise normal abdomens, but in the case of the chronic intestinal stasis such abnormalities, by common consent, are deemed more numerous.

Sir Arbuthnot Lane has no doubt at all that such "bands" and "membranes" are of acquired origin, and are due in the main to the erect posture of man. Lane applies very sound mechanical principles to his theories, and assumes that gravity itself is the determining factor in the causation of prolapse, and that nature, in her endeavour to prevent this calamity, not only thickens such suspensory ligaments as are normally present, but also provides subsidiary ligaments at points in the mesentary where maximum traction of the heavy loaded bowel exists; such ligaments he terms the "crystallisation of lines of force." Lane took a very general and logical view of his theory, and has supported it by analogy with other diseases, and by numerous examinations of the fœtal abdomen.

Thus he cites the pathological example of the "miner's elbow," a condition which in course of years results in a definite limitation of extension of the joint.

### The Developmental Theory.

Professor Arthur Keith, F.R.S., who is probably the greatest British expert in the subjects of morphology and embryology, does not consider these "bands" to be the definite result of chronic intestinal stasis, and finds that accessory bands, once regarded as pathological, are actually present in no less than 30 per cent. of newly-born children. He admits, however, that adhesions are largely increased in number and extent by pathological states. In other words, pathological varieties are exaggerations of normal adhesions or bands.

Keith also differs from Lane in supposing that such bands are the actual cause of chronic intestinal obstruction.

### Do Adhesions Cause Obstruction?

Mr. Smith says that in spite of the adhesions, accessory bands, the angulation and prolapse of the colon, he agrees with Keith when he states that "he has never yet obtained a single specimen which showed the usual signs of chronic obstruction—namely, marked constriction at the site of the adhesion with hypertrophy of the muscular coat of the intestine, which had to force its contents through the constriction."

At the present time all the evidence would seem to point to stasis being due not to mechanical obstruction, but to defect in the motor mechanism of the large intestine.

Keith attributes the control of rhythmic action in the gastro-intestinal canal to nodal tissue, situated in the myenteric or Auerbach's plexus, containing certain cells muscular in origin, with processes directly united with nerve cells on one hand and muscular cells on the other, having a higher degree of excitability than ordinary muscle and providing centres at which contracting waves take origin. It resembles, says Professor Keith, "the traffic along a railroad track, divided into sections, each placed under a pacemaker."

### Migratory Changes in the Colon.

In entering the field of discussion Mr. Smith supports the views of Professor Keith rather than those of Sir Arbuthnot Lane, and considers the abnormalities we have mentioned above to be primarily developmental in origin and due to one of two main causes—either failure of union of certain peritoneal surfaces, or persistence of certain peritoneal bands present during intra-uterine life, which usually disappear just before or just after birth. He continues:

"By reason of the migratory changes of position of the developing proximal colon, by the rotation of the U loop of the hind-gut at the second

**Chronic Intestinal Stasis**—(*continued*).

month of fœtal life and the anchorage of its anterior limb in front of the right kidney, by the subsequent descent of the cæcum from that position about and after birth, it will be apparent that the right side of the peritoneal cavity must be liable to such developmental changes. Thus if the cæcum and ascending colon fail to unite with the parietal peritoneum of the posterior abdominal wall, the mobile cæcum results."

Such reasoning is very sound and is supported by the author's researches in both the fœtal and adult abdomens; we must not forget, however, that the researches of Lane were just as complete, and with quite a different result. The correct origin of the causative factors of chronic intestinal stasis, as the reader will doubtless gather, is still "sub judice," although the balance of evidence put before us inclines towards a belief in the developmental theory. In any case, the profession owes a debt of gratitude to Sir Arbuthnot Lane for his genius in the pioneer investigations of this subject.

### Symptomatology of the Disease.

Be the origin of this disease what it may, there can be no doubt about its cardinal symptoms, and these may be briefly described under three headings: Pain, Constipation, and Indigestion.

Pain, which usually has a definite starting-point, is confined to the lower abdominal and lumbar regions, and is more frequently described on the right side than the left, in which case the symptoms suggest a sub-acute appendicitis, but *without* pyrexia: when such cases are sent for surgical advice there is usually a tender spot at the original site of pain.

Constipation, as the patient understands it, must not be taken as a synonym for intestinal stasis, as although frequent it is not constant, but is always more marked after the commencing or determining attack.

Indigestion is manifested by the existence of gastric or duodenal trouble, sometimes accompanied by epigastric pain.

The cause of this pain is somewhat obscure, and is thought by some authorities to be due solely to a reflex action. Sir Arbuthnot Lane is convinced that such symptoms are due to a mechanical factor by direct drag on the stomach and the second part of the duodenum by the prolapsed and laden colon. In support of his views Sir Arbuthnot has exhibited cases in which a colectomy has resulted in the cure of a duodenal ulcer.

We may mention in passing that Sir Arbuthnot Lane considers chronic intestinal stasis to be the root cause of most of the ills to which "the flesh is heir." Thus he would have it that exophthalmic goitre, tuberculosis, cancer, and a host of other diseases are caused by this condition. Whether he is right or not we are not at present in a fit state of knowledge to say with any honesty, but there can be no doubt that exophthalmic goitre, trigeminal neuralgia, and other diseases have been definitely relieved or cured by his operations, and it is only time which can show if his results are permanent and not sporadic.

### A Fascinating Theory.

If only all Sir Arbuthnot Lane's views on the effects of chronic intestinal stasis were proved to be true, a delightful vista of successful treatment would be extended before our eager eyes, for the possibility of the removal of the colon in the premonitory stages of disease might well be contemplated. Thus we may view the continued investigations of surgeons, physicians, and radiographers, such as Mr. Smith, Dr. Nathan Mutch, and Dr. Jordan with the greatest satisfaction, and we shall doubtless gather fresh information of value from their separate researches.

### Treatment.

This may be divided into medical or palliative treatment for the milder cases, and the surgical operations of "short-circuiting" or colectomy for the more advanced.

Medical treatment consists of the supporting or developing of the abdominal musculature by massage, exercises, or the wearing of a suitable abdominal support; this being combined with the internal administration of paraffin in order to lubricate the intestinal canal, and to secure a daily efficient evacuation of the bowels. Paraffin is a true laxative and not an aperient, and can thus be taken daily over long periods without any great inconvenience.

The surgical operations are lengthy, arduous, sometimes fatal, and not devoid of post-operative complications. In "short-circuiting" the intestine, the terminal ileum is connected with the lowest portion of the pelvic colon by means of a lateral or "end to end" anastomosis.

The disadvantage of such an operation is its unfortunate occasional sequela of regurgitation of the ileal effluent back into the descending and transverse colon.

Colectomy consists of the removal of the whole colon, and the anastomosis of the ileum to the terminal pelvic colon; such an operation is a lengthy one, results in a good deal of shock, and unfortunately often leads to multiple adhesions.

Against these primary disadvantages we have evidence of many cures from the symptoms of chronic intestinal stasis.

### Conclusions.

As indicated, the origin of the disease is still obscure, and the symptoms vary widely in different types of cases; indeed, the question of what actually constitutes chronic intestinal stasis has never yet been accurately defined, or its exact relation to toxic absorption determined. The treatment of the disease is not an ideal one, for it fails altogether in some cases, and has unfortunate sequelæ in others. Nevertheless, colectomy has proved its value in many cases, and is the best treatment extant for the cure of chronic intestinal stasis.

